# Diversity of Endonuclease V: From DNA Repair to RNA Editing

**DOI:** 10.3390/biom5042194

**Published:** 2015-09-24

**Authors:** Isao Kuraoka

**Affiliations:** Division of Chemistry, Graduate School of Engineering Science, Osaka University, 1-3 Machikaneyama, Toyonaka, Osaka 560-8531, Japan; E-Mail: kuraoka@chem.es.osaka-u.ac.jp; Tel.: +81-6-6850-6240; Fax: +81-6-6850-6240

**Keywords:** deamination, endonuclease V, DNA repair, RNA editing

## Abstract

Deamination of adenine occurs in DNA, RNA, and their precursors via a hydrolytic reaction and a nitrosative reaction. The generated deaminated products are potentially mutagenic because of their structural similarity to natural bases, which in turn leads to erroneous nucleotide pairing and subsequent disruption of cellular metabolism. Incorporation of deaminated precursors into the nucleic acid strand occurs during nucleotide synthesis by DNA and RNA polymerases or base modification by DNA- and/or RNA-editing enzymes during cellular functions. In such cases, removal of deaminated products from DNA and RNA by a nuclease might be required depending on the cellular function. One such enzyme, endonuclease V, recognizes deoxyinosine and cleaves 3' end of the damaged base in double-stranded DNA through an alternative excision repair mechanism in *Escherichia coli*, whereas in *Homo sapiens*, it recognizes and cleaves inosine in single-stranded RNA. However, to explore the role of endonuclease V *in vivo*, a detailed analysis of cell biology is required. Based on recent reports and developments on endonuclease V, we discuss the potential functions of endonuclease V in DNA repair and RNA metabolism.

## 1. Introduction

Although genomic DNA is supposed to contain error-free genetic information to facilitate the proper functioning of the cell, it is prone to damage, deterioration, and modifications due to environmental and endogenous factors. The induced DNA lesions interfere with DNA synthesis during replication and with RNA synthesis during transcription, leading to mutations and cell death. As a result, these mutations in the genome and subsequent disruption of cellular processes may cause cancer, congenital diseases, and aging. To maintain genomic integrity, cells possess several DNA repair pathways such as nucleotide excision repair (NER), which operates primarily on bulky helix-distorting damages caused by UV irradiation or genotoxic chemicals, and base excision repair (BER), which is used for non-bulky and non-helix-distorting DNA modifications induced by alkylation, oxidation, and deamination. DNA repair systems are highly conserved from bacteria to humans [1,2].

**Figure 1:**
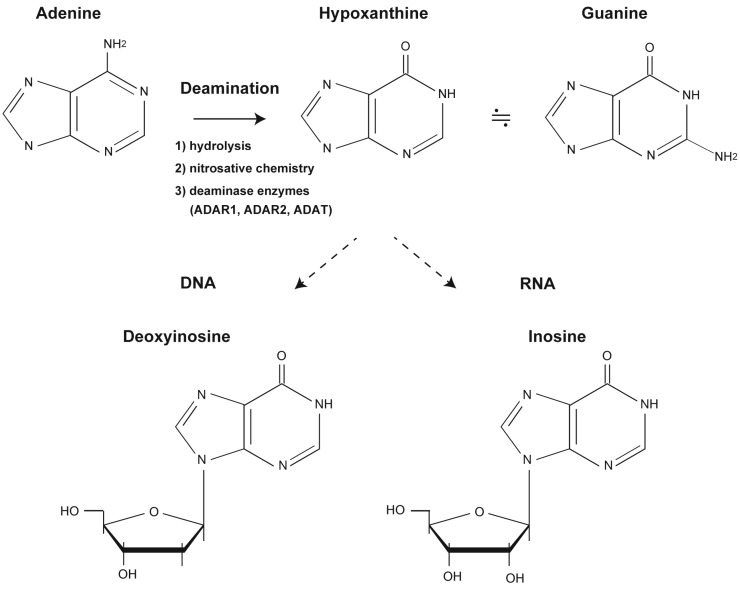
Formation of deoxyinosine and inosine. Deamination of adenine to hypoxanthine results in the formation of deoxyinosine in DNA and inosine in RNA. Hypoxanthine, which is recognized as guanine, pairs with cytosine. Deoxyinosine and inosine are nucleosides that form when hypoxanthine is attached to a deoxyribose ring or ribose ring, respectively.

Deamination of bases in nucleic acid strands is a spontaneous event induced by natural hydrolysis and nitrosative stress upon exposure to endogenous and/or exogenous agents (Figure 1). In humans, deamination events are thought to be stimulated by nitric oxide and superoxides produced in macrophages during inflammation. Deamination products that can pair with original bases can be potentially mutagenic during replication [3,4]. For example, the cytosine deamination product deoxyuridine is a nucleoside with uracil attached to the deoxyribose ring; this deamination product is subjected to BER, initiated by a monofunctional uracil-DNA glycosylase. This enzyme searches the genome to locate sites of damage and catalyzes the hydrolysis of the *N*-glycosidic bond to release the lesion and generate an apurinic/apyrimidinic (AP) site. Downstream enzymes subsequently process this AP site and use the information from the undamaged strand to restore the original coding sequence [5,6].

In the case of deoxyinosine, which is formed when hypoxanthine attaches to a deoxyribose ring formed by the deamination of adenine, BER is initiated by a monofunctional methylpurine DNA glycosylase. In addition, it is also subjected to the alternative excision repair (AER) pathway, which is biochemically distinct from NER and BER [7,8,9]. While NER involves dual excision of sites flanking the damaged base and BER involves the cleavage of the *N*-glycosidic bond between the damaged base and its sugar residue, AER pathway involves the initiation of a nick on the DNA strand, strictly on one side of the damaged base. The most extensively studied AER pathway is that of the *E*. *coli* endonuclease called endonuclease V (eEndoV). The enzyme, a product of the *nfi* gene, is highly conserved from bacteria to mammals suggesting that the human EndoV (hEndoV) repair pathway may play a key role in maintaining genome integrity and in cancer pathophysiology [7,8,10,11].

A recent report has shown that hEndoV localizes to the cytoplasm, preferentially binds to RNA, and cleaves the single-strand region containing inosine, which was generated by RNA-editing enzymes [12,13]. These data suggest the possibility that hEndoV controls the fate of inosine-containing RNA in humans, although its function as a DNA repair enzyme cannot be ruled out [14].

## 2. Deoxyinosine in DNA

**Figure 2:**
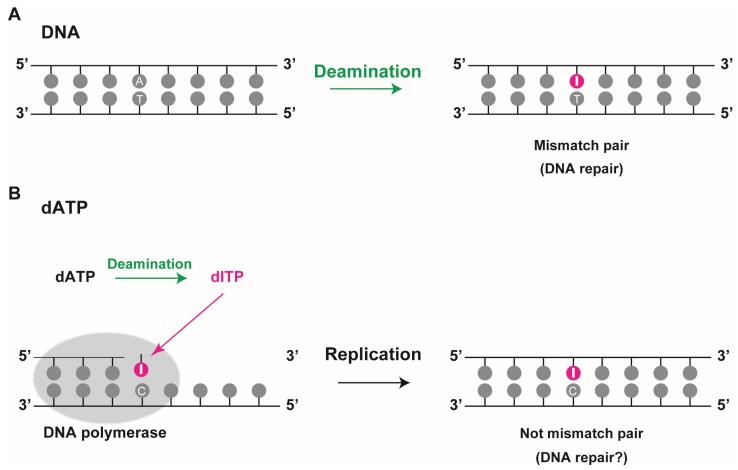
Deoxyinosine in DNA. (**A**) Deoxyinosine is produced by deamination of adenine in DNA. In this case, it results in a mismatched I:T pair that requires repair; (**B**) Deamination of dATP to dITP leads to its incorporation into DNA by DNA polymerase during replication. When deoxyinosine is recognized as guanine, it pairs with cytosine, resulting in a non-mismatch I:C pair.

Two fundamental mechanisms contribute to the generation of deoxyinosine in DNA (Figure 2). Under physiological conditions, deamination of DNA bases can occur spontaneously at a significant rate via a hydrolytic and nitrosative reaction in a pH- and temperature-dependent manner. Three of the four DNA bases, cytosine, adenine, and guanine, have amino groups and their deamination generates the corresponding base analogs uracil, hypoxanthine, and xanthine, respectively. While adenine deamination (resulting in the formation of deoxyinosine) in single-stranded DNA occurs under physiological conditions *in vitro* at a rate much lower than that of cytosine deamination[15], the rate of adenine deamination in double-stranded DNA (dsDNA), induced by nitrous acid, is similar to that of cytosine deamination [16]. A LC/MS-MS quantification of damaged DNA products revealed that deoxyinosines could be detected at background levels of 1–10 per 10^6^ nucleotides in tissues or cells [17]. Studies in *E*. *coli* and *Saccharomyces cerevisiae* also showed similar background levels of deoxyinosine (1.2 and 2.0 per 10^6^ nucleotides, respectively) [18]. These data suggest that deoxyinosines are spontaneously present, albeit in limited quantities. Though deoxyinosine in DNA cannot properly pair with thymine, it can bond well with cytosine, which in turn can result in an A to G transition mutation during DNA replication [19,20]. Therefore, deoxyinosine into DNA should be repaired so that the genome integrity is maintained (Figure 2A).

The second mechanism incorporates deoxyinosine triphosphate into the DNA during replication, since bases can be deaminated not only in the DNA strands but also in its precursor forms *i*.*e*., nucleotides (Figure 2B). Deamination of DNA precursors can lead to accumulation of non-canonical nucleoside triphosphates namely deoxyinosine triphosphate (dITP), deoxyxanthosine triphosphate (dXTP), and deoxyuridine triphosphate (dUTP) in the cell’s nucleotide pool. These can be incorporated into newly synthesized DNA, albeit less efficiently than regular DNA precursors. Pang *et al*. reported that mutation of inosine triphosphate pyrophosphatase (ITPase), an enzyme that hydrolyzes inosine triphosphate (ITP) and dITP, in *E*. *coli* and *S*. *cerevisiae* leads to a substantial increase in levels of deoxyinosine in the genome [18]. Further, ITPase*-*knockout mice also showed the incorporation of deoxyinosine into the mice genome [21]. Interestingly, the incorporation of deoxyinosine into DNA in *E*. *coli* results in non-mutation [22]. As deoxyinosine can pair with adenine, thymine, and cytosine, its mutagenic capability is expected to be high during DNA replication. However, since it is recognized as deoxyguanosine during DNA synthesis and can pair stably with cytosine via a nearly perfect hydrogen bond (I·C pair), in this context, it cannot lead to a mutation during replication. The incorporation of deoxyinosine during replication might not lead to conventional mismatch pairs such as a G·T pair in DNA. In higher organisms, high accumulation of deoxyinosine in DNA might generate single-strand breaks by the action of DNA repair enzymes such as endonuclease V and alkyl-adenine DNA glycosylase (AAG) [23,24]. Thus, in these cases, incorporation of deoxyinosine in the DNA increases the possibility of the development of DNA lesions that interfere with genome integrity.

## 3. Endonuclease V in DNA Repair

**Figure 3:**
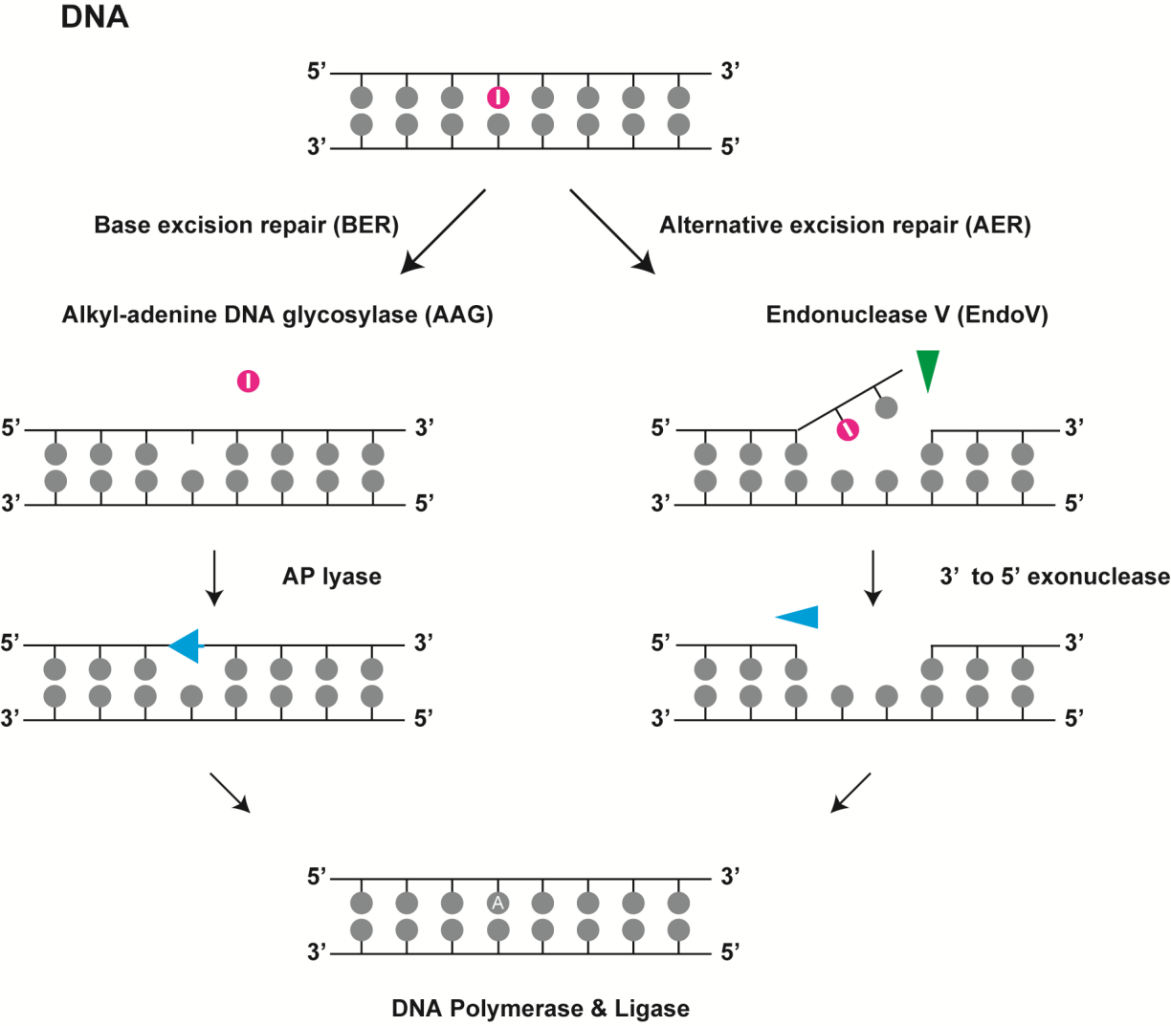
Endonuclease V in DNA repair. Deoxyinosine in DNA is repaired by alkyl-adenine DNA glycosylase (AAG) in the base excision repair (BER) pathway and by endonuclease V (EndoV) in the alternative excision repair (AER) pathway. EndoV hydrolyses the second phosphodiester bond located 3' to deoxyinosine in the DNA strand.

When deoxyinosine pairs with thymine, it should be repaired to protect the genome from further mutations. Both BER and AER pathways can perform this function (Figure 3). BER removes the damaged base moieties with a relatively small change in the chemical structure. DNA glycosylase recognizes the lesions and cleaves their glycosidic bonds. Recognition of the lesion is primarily accomplished by alkyl-adenine DNA glycosylases (*E*. *coli* AlkA; *S*. *cerevisiae* MAG; *H*. *sapiens* AAG), which not only remove the hypoxanthine base from DNA [23] but also recognize a broad range of purines altered by alkylation and oxidative damage including 3-mathlyadenine, 7-methylguanine, and *N*^6^-ethenoadenine [24].

An alternative DNA repair mechanism is the AER pathway, which only produces a single nick on one side of the damaged base and does not directly remove deoxyinosine from the DNA strand. eEndoV was identified as a repair enzyme that acts on sites of damage in double-stranded DNA such as AP sites, uracil, and UV-induced DNA lesions [25,26,27]. In subsequent experiments with a purified enzyme, eEndoV was found to be a deoxyinosine 3' endonuclease that recognizes deoxyinosine in both dsDNA and single-stranded DNA (ssDNA) and cleaves the second phosphodiester bond 3' to the mismatched I·T pair, leaving a nick with 3' hydroxyl and 5' phosphate groups [28]. Studies carried out using a *nfi* mutant strain have revealed a high frequency of A·T to G·C transitions following exposure to nitrous acid, leading to deamination and accumulation of deoxyinosine in DNA [20]. This enzyme has subsequently been shown to have a broad substrate spectrum that includes AP sites, urea residues, base mismatches, flap DNA, pseudo Y structures and loops, and hairpins [28,29,30]. Since it cannot release deoxyinosine or other damaged bases, eEndoV is thought to require another enzyme to complete the repair process. Lee *et al*. reported that eEndoV, 3'-exonuclease activity of DNA polymerase I, and DNA ligase are sufficient to remove deoxyinosine from DNA [10]. In a model of *E*. *coli*, an EndoV-dependent AER pathway for the removal of deoxyinosine from DNA has been identified. However, the AER pathway has not yet been elucidated in mammalian cells. According to a biochemical investigation on mammalian EndoV, it possesses considerable deoxyinosine 3' endonuclease activity in deoxyinosine-containing ssDNA [7,12,13]. However, this activity is very poor in dsDNA, thus suggesting that it requires another enzyme for effective cleavage in this case. Until now, no additional enzyme has been found.

These two pathways might be employed based on the cellular conditions or external environment such as exposure to nitric oxide or S phase DNA replication. For instance, while DNA glycosylases might repair the deoxyinosines on dsDNA in the G2/M phase, mammalian EndoV may act on deoxyinosines in the ssDNA region of replication fork during DNA synthesis. There could be some redundancy in the repair capacity for deoxyinosine.

It is to be noted that endonuclease V is different from T4 endonuclease V, a bifunctional DNA glycosylase encoded by the bacteriophage T4 DNA genome, which hydrolyzes the *N*-glycosidic bond of the 5' end to UV-induced cyclobutane pyrimidine dimers and cleaves the 3' end of the resulting AP site [31,32].

## 4. Inosine in RNA

**Figure 4:**
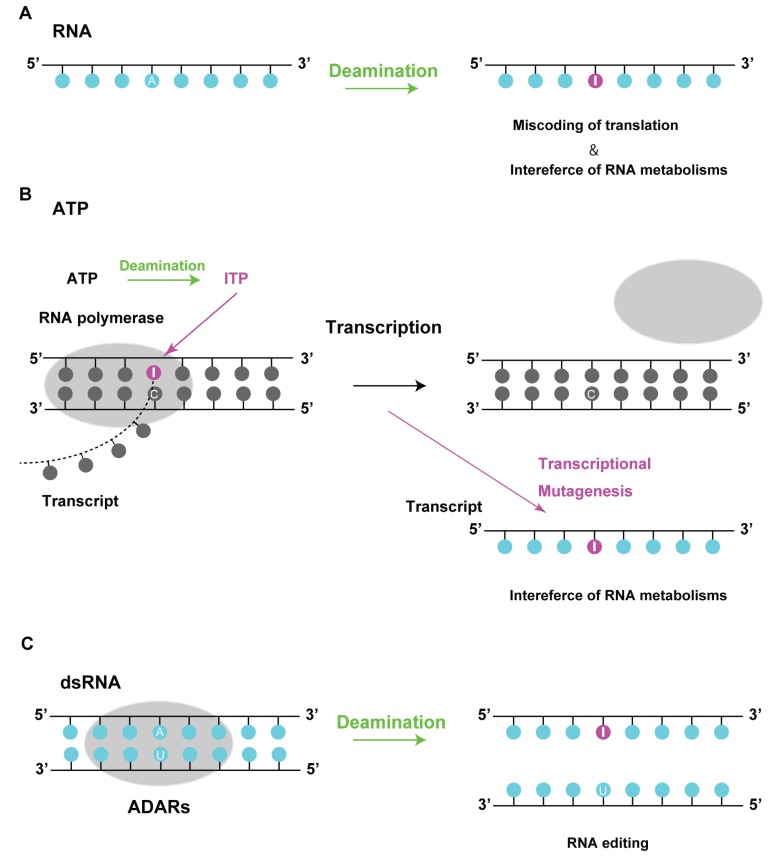
Inosine in RNA. (**A**) Inosine is produced by deamination in RNA. As inosine is recognized as guanine during translation, this deamination leads to miscoding of transcription or interference of RNA metabolism; (**B**) Deamination of ATP produces ITP, which accumulates in the nucleotide pool and is inserted into the transcript by RNA polymerase during transcription elongation. Transcriptional mutagenesis results in miscoding of proteins or interference of RNA metabolism; (**C**) Conversion of adenosine (A) residues to inosine (I) within dsRNA is catalyzed by adenosine deaminases acting on RNA (ADARs). They catalyze RNA editing at specific sites in the dsRNA structure, suggesting that their proper functioning may be crucial to RNA metabolism.

Three fundamental mechanisms contribute to the presence of inosine in RNA (Figure 4). One is a spontaneous and incidental modification effected by means of a hydrolytic and nitrosative reaction (Figure 4A) [15,33]. Quantification analyses in *E*. *coli* and *S*. *cerevisiae* revealed that inosines in RNA could be detected at background levels of 11 and 42 per 10^6^ nucleotides, respectively [18]. These levels seem to be higher than that of deoxyinosines in DNA, suggesting that inosine is one of the common modifications incorporated into tRNA [18,34]. Elevated temperatures at pH 7.4 have been shown to induce the conversion of adenine to hypoxanthine in ssDNA [15,33], and this could be the case in mRNA as well. Nitrosative stress caused by nitric oxide-derived nitrous anhydride increases during inflammation, leading to the production of hypoxanthine from adenine.

Another mechanism is the unexpected incorporation of ITP into the mRNA by RNA polymerase during transcription elongation (Figure 4B). Although ITP is removed by an inosine triphosphatase (ITPase) from the cellular nucleotide pool, incorporation of ITP is caused by an imbalanced purine nucleotide metabolism. When there are conditions that dITP can be produced by deamination of deoxyadenosine triphosphate (dATP), ITP can also be produced by deamination of adenine. Like DNA polymerase, RNA polymerase can insert ITP into transcripts during transcription elongation. Defects in purine nucleotide metabolism lead to incorporation of inosine into RNA in *E*. *coli* and *S*. *cerevisiae* [18]. In addition, ITPase-deficient mice and IDPase-knockdown cells show the accumulation of inosine in RNA [21], indicating that excessive generation of ITP by deamination may lead to assimilation of inosine lesions during RNA synthesis.

Inosines incorporated in mRNAs via spontaneous and incidental deamination are thought to be potentially mutagenic since inosine is recognized as guanine during translation and thus could generate mutant proteins that negatively affect cell viability. In addition, presence of inosine in mRNA via transcription and in tRNA via these two mechanisms may interfere with RNA metabolism.

The third mechanism is the conversion of adenosine to inosine by the RNA-editing enzymes adenosine deaminases, acting on RNA (ADAR) and tRNA (ADAT) (Figure 4C) [35,36,37]. Though three ADARs (ADAR1, ADAR2, and ADAR3) have been described in mammals, only ADAR1 and ADAR2 have proven catalytic activity, but ADAR3 has not demonstrated. Two major isoforms of ADAR1 exist in mammalian cells; the p110 form is constitutively expressed and predominantly nuclear, while the p150 form is inducible by interferon and shuttles between the nucleus and the cytoplasm [38]. ADAR2 is highly expressed in the brain and localizes to the nucleoli and cytoplasm [39]. These enzymes predominantly catalyze RNA editing at specific sites in the dsRNA structure, suggesting that ADAR-mediated RNA editing may be important for gene expression and gene control [35,37,40]. ADARs are thought to hyperedit long dsRNA, which can result in up to 50% of A residues being changed to I residues [40,41]. This can result in codon changes since inosine is interpreted as guanosine by the translation machinery. Moreover, hyperedited inosine-containing dsRNA is likely to have localized changes in its RNA structure owing to the relative instability of IU pairs, which in turn cause the unwinding of its helices.

In tRNA, inosine occurs at the wobble position of anticodons. Since it is postulated that these tRNAs can translate three different codons ending with U, C, or A, the presence of inosine in the anticodon region of tRNA is essential for viability [42]. ADATs can catalyze this editing to expand their coding capacity. For example, ADAT1 in *S*. *cerevisiae* can convert adenosine to inosine at position 37, which is beside the anticodon loop. Members of the ADAT family are conserved across organisms ranging from *E*. *coli* to *H*. *sapiens*. ADATs have been hypothesized to be the evolutionary ancestors of ADARs [36]. In addition, certain microRNAs and endogenous short interfering RNAs are edited by ADARs [43]. Taken together, it can be assumed that the presence of inosine in RNA is one of the key modifications in RNA and should ideally not lead to miscoding during translation or cause interference in RNA editing that may disrupt downstream cellular activities.

## 5. EndoV in RNA Editing

**Figure 5:**
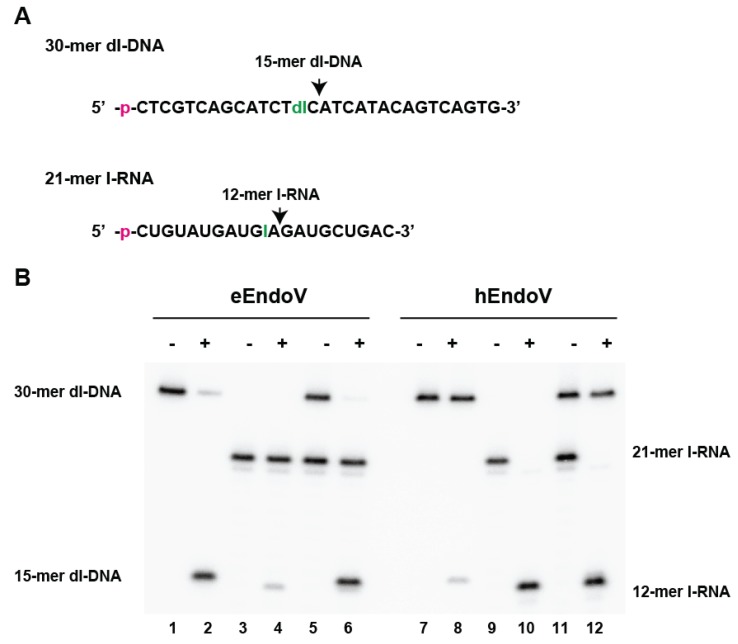
*E*. *coli* endonuclease V (eEndoV) and human endonuclease V (hEndoV) display opposing preferences in cleaving RNA and DNA. (**A**) ^32^P-labelled 30-mer ssDNA containing deoxyinosine and 21-mer ssRNA containing inosine (dI and I, respectively) were used as substrates. The positions of cleavage are indicated by arrows; (**B**) Activity of eEndoV (lanes 1–6) or hEndoV (lanes 7–12) when incubated with ^32^P-labelled DNA substrate (30-mer dI-DNA) containing deoxyinosine (lanes 1–2 and lanes 7–8) or ^32^P-labelled RNA substrate (21-mer I-RNA) containing inosine (lanes 3–4 and lanes 9–10) or both DNA and RNA substrates (lanes 5–6 and lanes 11–12) at 37 °C for 30 min. The cleavage products (15-mer dI-DNA and 12-mer I-RNA) are indicated by denaturing polyacrylamide gel analysis.

Purified eEndoV and hEndoV recognize and cleave both dsDNA and ssDNA containing deoxyinosine, as well as ssRNA containing inosine. However, the efficiency of cleavage differs between DNA substrates containing deoxyinosine and RNA substrates containing inosine (Figure 5). While eEndoV cleaves ssDNA containing deoxyinosine more efficiently than it cleaves ssRNA containing inosine (Figure 5, lanes 1–6), hEndoV cleaves ssRNA substrates containing inosine better than it cleaves ssDNA containing deoxyinosine (Figure 5, lanes 7–12). Interestingly, hEndoV also does not effectively cleave dsRNA containing inosine [13]. This suggests that the main function of EndoV is organism-specific and dependent on intra- and extra-cellular environments.

When a spontaneously produced inosine in RNA is subjected to hEndoV nuclease activity, it functions to prevent the formation of mutant proteins. However, a ribonuclease that acts along with hEndoV to digest the cleaved ssRNA has not yet been identified. On the other hand, removal of inosine resulting from ITP incorporation during transcription suggests that hEndoV is possibly coupled with transcription and/or RNA processing mechanism because it is important to ensure that the mRNA transcript is mutation-free before translation.

**Figure 6:**
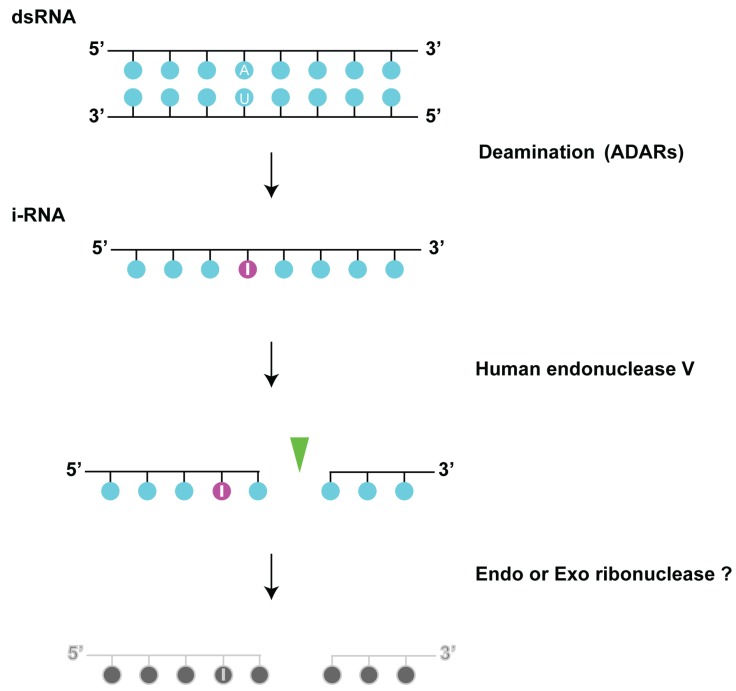
Human endonuclease V in RNA editing. Inosine in RNA is produced by ADARs or inosine monophosphate (IMP). Human endonuclease V can cleave the second phosphodiester bond located at 3' end of inosine in the RNA strand. Endo- or exo ribonuclease will further digest the cleaved RNA.

Conversion of adenosine residues to inosine within dsRNA is catalyzed by ADARs (Figure 6). As hEndoV cleaves at a locally distorted ssRNA structure containing inosine, it might be able to act on partially unwound dsRNA hyperedited by ADAR1, given that it could cleave dsRNA treated by ADAR2 [13]. hEndoV cleaves ssRNA containing inosine but not dsRNA; therefore, might require ADARs that have dsRNA unwinding activity. Thus, hEndoV might participate in the degradation of edited dsRNA (e.g., long viral dsRNA genome). In addition, hEndoV cleaves inosine at a biologically important site for deamination in the transcript of a neurotransmitter [44,45,46], suggesting that this enzyme is involved in normal RNA metabolism.

## 6. Conclusions

Alignment of the amino acid sequence of EndoV homologs revealed the following characteristics for hEndoV and eEndoV: identities = 74/199 (37%), positives = 108/199 (54%), and gaps = 19/199 (10%) [13]. These enzymes are highly conserved from bacteria to humans. However, there seems to be a difference in substrate specificity between eEndoV and hEndoV. According to genetic studies and biochemical results in *E*. *coli*, eEndoV is thought to be a DNA repair enzyme in the AER pathway, which functions to remove deoxyinosines from deaminated DNA. In humans, hEndoV prefers ssRNA containing inosine over ssDNA containing deoxyinosine. These data indicate that hEndoV possesses an RNA-editing function that could control RNA quality (mRNA, tRNA, microRNAs and so on). Nevertheless, we cannot exclude the possibility that post-translational modifications on hEndoV could enhance its nuclease activity towards DNA. To understand the physiological functions of hEndoV in entirety, extensive analyses of cell biology might be required. Importantly, investigations on the cellular function of inosine in RNA are necessary.

Basically eEndoV in bacteria and hEndoV in mammals were discussed in here. It is to be noted that *Pyrococcus furiosus* EndoV (PfuEndoV) in the archaeal cells is purified and characterized [47]. Interestingly, this enzyme can cleave dsDNA containing deoxyinosine and ssRNA containing inosine. It is not clear yet whether PfuEndoV in the archaeal cells possess only DNA repair but also an RNA-editing. Further study of EndoV function in three domains of living organisms might lead to finding of new cell functions.
